# Life-Threatening Thrombocytopenia Secondary to Trimethoprim/Sulfamethoxazole

**DOI:** 10.7759/cureus.1963

**Published:** 2017-12-19

**Authors:** Pramod Gaudel, Ahmed H Qavi, Prasanta Basak

**Affiliations:** 1 Department of Medicine, Albert Einstein College of Medicine, Montefiore Medical Center, New York, United States

**Keywords:** platelets, tmp/smx, thrombocytopenia, bleeding

## Abstract

Thrombocytopenia is an uncommon side effect of trimethoprim/sulfamethoxazole (TMP/SMX) when given in the usual recommended adult dosage. We report a case of severe and possibly life-threatening thrombocytopenia associated with TMP/SMX therapy.

A 92-year-old female presented after a mechanical fall and subsequent intractable bleeding from a laceration on her left leg. She had a history of cellulitis of the lower extremities treated with a 10-day course of TMP/SMX. Her last dose was two days before the visit. The physical examination was significant for a small laceration on her left shin, with persistent oozing of blood. Her blood work was notable for white blood cells (WBC) 9.4×10^9/L (9.4×10^3/mm^3), hemoglobin 125g/L (12.5 g/dL) and platelets 5×10^9/L (5×10^3/mm^3). A repeat platelet count was 4×10^9/L. Prothrombin time was 11 seconds and the international normalized ratio (INR) was one. The TMP/SMX was discontinued and one unit of platelets was transfused. Her platelet count subsequently increased to 108×10^9/L.

Severe thrombocytopenia with a platelet count of ≤10×10^9/Lmay rarely result in the catastrophic spontaneous bleeding. Thus, low platelet counts associated with TMP/SMX carry potential life-threatening complications. The clinicians should be aware of this adverse effect of TMP/SMX, which appears to be dose/duration independent. We suggest careful monitoring of complete blood cell count, especially platelet count, before and during TMP/SMX therapy.

## Introduction

Trimethoprim/sulfamethoxazole’s (TMP/SMX) antibacterial activity is used in treating gram-positive and gram-negative infections as well as in the prophylaxis and treatment for Pneumocystis carinii pneumonia. Thrombocytopenia is an uncommon side effect of TMP/SMX. When given in the usual recommended adult dosage, its estimated excess risk is 38 cases per one million users per week. The severity of thrombocytopenia with TMP/SMX is usually mild-to-moderate [1]. We report a case of severe and possibly life-threatening thrombocytopenia associated with TMP/SMX therapy.

## Case presentation

A 92-year-old female presented to the emergency department with intractable bleeding from a laceration on her left leg, following a mechanical fall. She denied any rash, bruising or bleeding from the gums or other orifices. She had a history of hypertension, hyperlipidemia and chronic leg edema with recurrent cellulitis. Her home medications included nebivolol, Torsemide, losartan, atorvastatin, and oxybutynin. On examination, the vital signs were stable. The physical examination was unremarkable except for a small laceration on her left shin, with constant oozing of blood. No purpura or mucosal hemorrhage was noted. The stool guaiac test was negative. Complete blood count (CBC) revealed white blood cells (WBC) 9.4×10^9/L (9.4×10^3/mm^3), hemoglobin 125g/L (12.5 g/dL) and platelets 5×10^9/L (5×10^3 /mm^3). A repeat platelet count was 4×10^9/L. Prothrombin time was 11 seconds and the international normalized ratio (INR) was one. The blood urea nitrogen and creatinine were 26.07 mmol/L (73 mg/dL) and 435.91 mmol/L (4.93 mg/dL) respectively. Table 1 shows the patient’s CBC over the course of her hospitalization.

**Table 1:** The complete blood count before and during hospitalization. WBC: white blood cell count, Hb: hemoglobin.

Complete blood count (CBC)								
Hospital day	Baseline (1 month ago)	1	1 (Repeat)	2	3	4	5	10
WBC (x10^3^/mm^3^)	8.8	9.4	8.5	9.5	5.8	6.4	7	8.2
Hb (g/dL)	12.3	12.5	11.6	10.7	10.1	10.2	10.6	11
Platelet( x 10^3^/mm^3^)	165	5	4	51	62	88	108	204

The peripheral blood smear showed severe thrombocytopenia with no platelet clumping and no evidence of fragmented cells or schistocytes to suggest thrombotic thrombocytopenic purpura. The serum lactate dehydrogenase, thyroid functions, and vitamin B12 levels were normal.

One month ago, the platelet count was 165×10^9/L and creatinine 79.58 mmol/L. It was later determined that two days prior to the admission, the patient had completed a 10-day course of TMP/SMX prescribed by her primary care physician for cellulitis of the lower extremities. The Naranjo algorithm score was 7, indicating a probable adverse drug reaction to TMP/SMX resulting in thrombocytopenia. The TMP/SMX was discontinued and one unit of platelets were transfused, with the increase in platelet count to 108×10^9/L. The patient’s renal function also improved with intravenous fluid (IV) fluids and discontinuation of the TMP/SMX. She was discharged on day three of admission.

## Discussion

Drug-induced thrombocytopenia (DITP) can be a result of three different processes: a) direct injury to the bone marrow consequently affecting its thrombopoietic function, b) immune-mediated process with the antibody production, and c) formation of hapten [1]. Thrombocytopenia associated with TMP/SMX is an immune-mediated process resulting in the platelet destruction by drug-dependent platelet antibodies. A patient presenting with acute thrombocytopenia (< 20,000/mm 3) of unclear etiology should warrant a strong suspicion of DITP. The usual history includes drug exposure of at least five-seven days in a first-time user [2]. We may also use a clinical criterion suggested by George, et al. to evaluate a patient suspected to have DITP [3]. Table 2 depicts the criteria. Our patient meets the criteria I, II and III indicating that the TMP/SMX was the probable culprit drug leading to thrombocytopenia. She was not rechallenged with the drug, hence the criteria IV was not met.

​

**Table 2:** The criteria and the level of evidence for assessing drug-induced thrombocytopenia. Refer citation [1,3].

Criteria #	Criteria consideration		
1	Therapy with the suspected drug was instituted prior to the development of thrombocytopenia and the resolution of thrombocytopenia occurred after the discontinuation of the suspected drug		
2	Only the suspected drug was used before the onset of thrombocytopenia and the platelet count was normal or continued to rise toward normal range with continuation or reinstitution of other drugs after the suspected drug was discontinued ​​​​		
3	Other causes for thrombocytopenia were excluded		
4	The suspected drug resulted in the recurrent thrombocytopenia on re-challenge or re-exposure		
Level of evidence		Criteria met	Remark
I		1, 2, 3 and 4	The suspected drug is a definite cause
II		1, 2 and 3	The suspected drug is a probable cause
III		1 only	The suspected drug is a possible cause
IV		1 is absent	The suspected drug is unlikely to be a cause

We sometimes use serological assays testing patient’s serum against normal platelets in the presence and the absence of the drug in question. The evidence of drug-dependent antibodies may help confirm the etiology. However, these tests are not routinely done due to their low sensitivities, unavailability in many medical centers, and due to the cost and time constraints [2].

**Figure 1:**
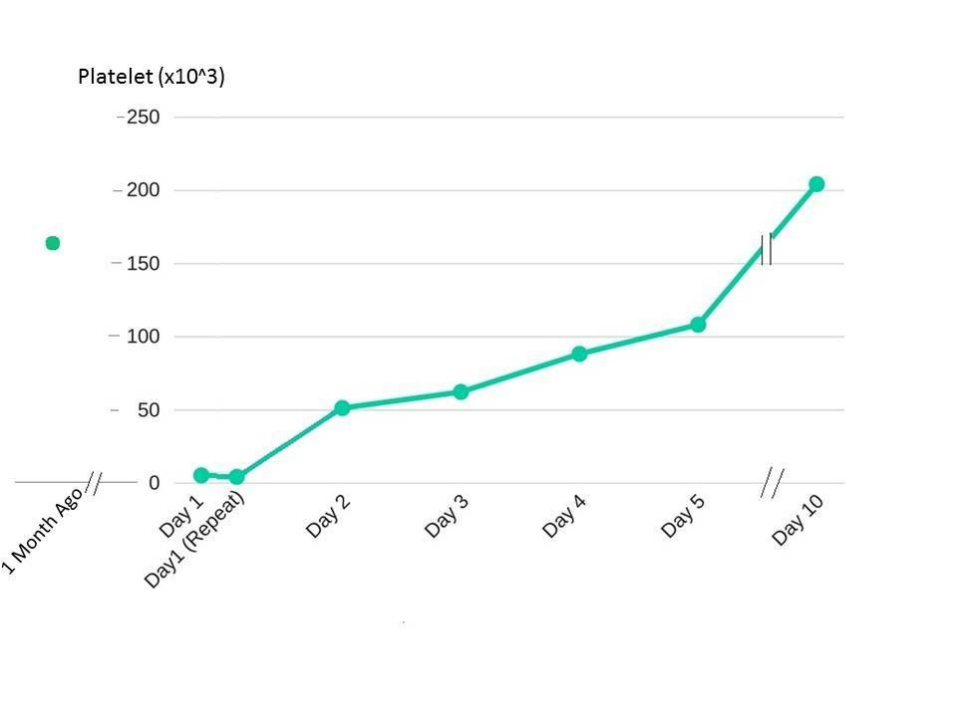
The patient's platelet trend before and after the discontinuation of trimethoprim/sulfamethoxazole.

The treatment of thrombocytopenia associated with TMP/SMX therapy includes discontinuation of the offending drug and the use of corticosteroids. In most circumstances, the withdrawal of the drug alone may be sufficient. The systemic corticosteroids when required, are highly efficacious in up to 90% of the cases due to their anti-inflammatory and thrombopoietic effects. The usual dosage is 0.3 to 2.0 mg/kg/day and the treatment may be continued for four to six weeks to obtain complete recovery [4-5]. In our patient, the corticosteroids were not needed and drug discontinuation along with the platelet transfusion resulted in the recovery (Figure 1).

The platelet transfusion and intravenous immunoglobulin (IVIG) may be required in some patients. The IVIG may be usually the second line of therapy, mostly due to its high cost and because corticosteroids are less expensive and more superior in efficacy. The IVIG may be used once corticosteroids have failed, in the patients with brittle diabetes due to fear of steroid-induced uncontrolled diabetes or in young children. The standard dosage is 0.4 g/kg daily for five days or one g/kg daily for two to three days. It is thought to act by saturating platelet receptors which in turn prevents binding of drug-induced antiplatelet antibodies, consequently decreasing the platelet destruction [5].

Severely low platelet counts of ≤ 10×10^9/L have been implicated in severe spontaneous bleeding on rare occasions. Thus, thrombocytopenia associated with TMP/SMX carries the potentially life-threatening complications. This hematologic adverse effect of TMP/SMX, which appears to be dose/duration independent, should warrant careful monitoring of complete blood cell count, especially platelet count, before and during TMP/SMX therapy [5].

## Conclusions

In an era where TMP/SMX is increasingly prescribed for possible community acquired methicillin-resistant Staphylococcus aureus (MRSA) infections, especially in the urinary and respiratory tracts, the physicians should be mindful of the rare adverse effect of life-threatening thrombocytopenia. The patients on TMP/SMX should, therefore, be closely observed for cutaneous manifestations and bleeding attributable to thrombocytopenia. Such standard of care would allow prompt withdrawal of the drug with subsequent recovery as seen in our patient.
